# Necrotizing fasciitis: treatment concepts & clinical outcomes – an institutional experience

**DOI:** 10.1186/s12893-024-02638-2

**Published:** 2024-10-28

**Authors:** Ajay Raveendranadh, S. S. Prasad, Vivek Viswanath

**Affiliations:** Department of Surgery, Kasturba Medical College, Manipal, Manipal Academy of Higher Education, Manipal, 576104 India

**Keywords:** LRINEC, Necrotizing fasciitis, NSTI, Procalcitonin

## Abstract

**Background:**

A severe infection of the skin and soft tissues, Necrotizing Fasciitis (NF), spreads quickly along the deep fascia. This study aimed to characterize the clinicopathological features, analyze the implicated bacteria’s antibiotic sensitivity, evaluate surgical management, and assess the diagnostic accuracy of the Laboratory Risk Indicator for Necrotizing Fasciitis (LRINEC) score in Necrotizing Soft Tissue Infection (NST).

**Methods:**

This single-center prospective observational study was conducted in the Department of General Surgery, Kasturba Medical College, Manipal, with 171 proven cases of NSTI between 2019 and 2021. Clinico-demographic data and laboratory investigation values were collected at two-time points (at admission and 72 h after admission). Imaging data, LRINEC score, culture results, and antibiotic sensitivity were recorded. Appropriate descriptive and analytical statistics were used for the statistical analysis.

**Results:**

Of the 171 patients, 150 were male (87.7%). The mean age was 57.6 ± 13.1 years. The presenting features in all the cases were pain, swelling, and fever. Diabetes mellitus (DM) is the most common comorbidity. The lower extremities were the most commonly affected sites. Streptococcus pyogenes showed significant growth in 25.41% of the samples. Ceftriaxone sensitivity was seen in 41/141. A score of ≥ 8 was obtained in 118/171 (69%) patients, suggesting a higher severity and significant risk for NSTI. The Area Under the Curve of Receiver Operating characteristic Curve (ROC) for establishing diagnostic accuracy for LRINEC was 0.694. Mortality was significantly higher in the patients with higher LRINEC scores and elevated procalcitonin. The mortality rate was higher in patients who underwent surgery within 12 h.

**Conclusion:**

Necrotizing fasciitis is a soft tissue infection with a high mortality rate. The clinical features and determinants of mortality in patients with NF are highlighted in this study. At the outset, a high index of suspicion was critical. Using prognostic evaluation techniques in daily clinical practice will assist medical professionals in providing adequate on-time care and significantly lowering mortality. The AUC for LRINEC score, although significant, is low. LRINEC score is not to be used to determine whether surgical intervention should be expedited or anticipated. Its role is to aid in prognosticating the outcome of the individual patient. Our study concludes that early extensive surgical debridement remains the single most crucial intervention in patients diagnosed with necrotizing fasciitis (NF), regardless of disease severity and the LRINEC score.

## Introduction

Necrotizing soft tissue infection (NSTI) is a unique, deadly, life-threatening disease that mostly destroys the fascial layer, deeper subcutaneous tissue, and sometimes even muscles [1]. Hippocrates described a clinical case of necrotizing fasciitis (NF), a consequence of erysipelas disease, around 500 BC. This description is similar to the modern understanding of NF [2]. According to reports, the prevalence of NSTI is 0.40 cases per 100,000 people worldwide, and its yearly incidence is predicted to be between 500 and 1,000 cases. This condition frequently affects middle-aged and older individuals. The 3:1 male-to-female ratio indicates a higher prevalence among men; this ratio is primarily associated with a higher incidence of Fournier’s gangrene in men [3].

The development of NF is linked to the combined effects of the host’s specific characteristics and bacterial virulence factors. Various bacteria, toxins, and enzymes produced promote the spread of infection and necrosis [4]. The prognosis is worse in the presence of coexisting conditions, including diabetes mellitus, immunodeficiency disorders, cirrhosis of the liver, or renal failure [5]. Although facultative anaerobic and aerobic organisms can induce NSTI, polymicrobial bacteria are the most common cause. Therefore, it is essential to diagnose NF immediately. Given its link to more extensive surgery, increased rates of amputation, and higher fatality rates, any delay might prove catastrophic. In addition, infection may cause Systemic Inflammatory Response Syndrome (SIRS) if treatment is not administered. Local pain, swelling, and erythema are the classic triad of symptoms that patients with NSTI typically present with. The most common abnormalities in vital signs were fever and tachycardia (> 100 beats/min), followed by hypotension (Systolic BP < 100 mmHg) and tachypnea (> 20/min). Skin erythema and these abnormalities are most helpful in differentiating NF from other soft tissue infections. The infected site exhibits tenderness, sclerosis, skin necrosis, and hemorrhagic bullae [6, 7]. Under these conditions, laboratory findings are often non-specific. Nonetheless, specific test results can assist physicians in distinguishing NSTI from other skin conditions. One such example is the Laboratory risk indicator for Necrotizing Fasciitis (LRINEC) proposed by Wong et al. (Table 1) [8]. It is a clinical tool that can be used as an adjunct in the clinical diagnosis of NF based on six common serum parameters: C-reactive protein (CRP), total white, cell count, haemoglobin, serum sodium, creatinine and glucose. Wong et al. suggest a LRINEC threshold of ≥ 6 for patients with a suspicion of NF and a score of ≥ 8 for patients with a strong prediction for the disease. While the LRINEC score is a useful tool, it cannot be used in isolation. A thorough clinical assessment, including the history, physical examination, and imaging modalities along with the LRINEC sore, improves the diagnostic accuracy of other NSTI.

**Table 1:** The LRINEC score serum parameters

Laboratory Risk Indicator for Necrotizing Fasciitis		
PARAMETER	RANGE	SCORE
Hb (g/dL)	> 13.5	0
	11-13.5	1
	< 11	2
WBC (10⁹/L)	< 15	0
	15–25	1
	> 25	2
Sodium (mmol/L)	< 135	2
Creatinine (mg/dL)	> 1.6	2
Glucose (mg/dL)	> 180	1
C Reactive Protein	> 150	4

A Score ≤ 5 = < 50% risk (low); 6–7 = 50–75% risk (moderate); ≥8 = > 75% risk (severe)

In addition to providing early disease detection, this score can aid patient categorization into risk groups and the distribution of diagnostic resources. Plain radiography can reveal the presence of gas within soft tissues despite having limited sensitivity and specificity. Compared with typical radiography, CT and MRI are more specific and sensitive. The degree of gas formation, inflammation, tissue edema, and tissue infection may all be observed on CT scans. Although MRIs is more expensive than CT, it provides more precision. Another practical alternative is ultrasonography, which can help determine the type and severity of infection, particularly in cases where the diagnosis is not entirely clear [9, 10]. Successful treatment requires a multidisciplinary approach to intensive care, with active fluid replacement and sepsis management, rigorous surgical debridement, and broad systemic antibiotic drugs [11].

A comprehensive literature review found a lack of information regarding NSTI in patients from India. In a tertiary center in southern Karnataka, this study aims to characterize the clinicopathological features and analyze the bacteria implicated in the antibiotic sensitivity, surgical management, and diagnostic accuracy of the LRINEC score in NSTI.

## Materials and methods

This single-center prospective observational study was conducted in the Department of General Surgery, Kasturba Medical College, Manipal, between December 2019 and September 2021. The study commenced after obtaining approval from the Institutional Ethics Committee (659/2019), and written informed consent was obtained from all patients. The inclusion criteria were met by patients with clinically diagnosed NSTI who were admitted to the Department of General Surgery during the study period, including those who were discharged against medical advice. Those patients who had not received primary treatment for NF at our institution were excluded from the study. This includes individuals who were diagnosed and treated, either medically or surgically, at another hospital and then sought further management at our facility. Two surgeons independently diagnosed NF. In cases where there was diagnostic ambiguity, a third independent surgeon’s opinion would be sought before treatment was initiated. Before surgery, an emergency radiograph of the affected region was taken for each patient. Clinical data, including a detailed history, clinical examination findings, and laboratory investigation values, were collected at two time points: one at the time of admission and the other 72 h after admission. Imaging data, LRINEC score, culture, and antibiotic sensitivity; data on surgical management, including the number of debridements, amputations (if any), and reconstructive surgeries were collected, and the duration of hospitalization was recorded. Patients were categorized as having a low (points ≤ 5; <50% probability for the presence of NSTI), medium (points 6–7; 50–75% probability for the presence of NSTI), or high risk (points ≥ 8; >75% probability for the presence of NSTI) of NSTI based on the LRINEC score results. This score should encourage early detection and distinguish it from other serious soft tissue infections that require alternative treatment approaches [12]. Emergency surgical debridement was planned for all patients. A daily dressing was applied to the wound until reconstructive surgery was performed. Across the surgical units, the dressing technique would utilize a 10% povidone-iodine aqueous solution to clean the affected area, followed by the application of sterile cotton dressings as the standard care. All dressings are changed at least once in 24 h. Pus and tissue samples were sent for culture sensitivity. As per the institutional antibiotic policy, patients diagnosed with NF across all surgical units were empirically started on injectable Piperacillin-Tazobactam and Clindamycin. It was later switched to therapeutic broad-spectrum antibiotics that were focused on culture sensitivity. Supportive therapy, such as blood product transfusion, blood sugar management, nutritional support, and fluid and electrolyte balance maintenance, was administered when necessary. In certain instances, resuscitation with intravenous fluids and colloids is required.

### Statistical analysis

Statistical analysis was performed using IBM SPSS (Statistical Package for Social Sciences) version 20. The analysis included the generation of frequency tables, bar and pie charts. The association of variables was assessed using the Chi-square test. If any cell frequency was < 5, Fisher’s exact test was used (for tables larger than 2 × 2). Continuous variables were reported as mean ± standard deviation (SD) or median (interquartile range), while categorical data were expressed as frequencies and percentages. The normality of data was assessed using the Shapiro-Wilk test.

Additionally, we conducted a receiver operating characteristic (ROC) analysis to estimate the diagnostic accuracy of the LRINEC score cut-off in NSTI. Differences in survival between patients with high and low LRINEC scores upon admission were analyzed using Kaplan–Meier analysis. Kaplan-Meier curves illustrated the survival in these patient groups, and log-rank tests were used to assess any differences.

All statistical tests were two-tailed, with a significance level of *p* < 0.05.

## Results

This study included all patients who satisfied the eligibility criteria during the study period. A total of 171 adult patients were included, with a mean age of 57.6 ± 13.1 years. The majority (55%) were aged 51–70 years, with a male-to-female ratio of 50:7. Clinical characteristics included signs and symptoms at the site of involvement, and the vitals observed. Other clinicopathological tables have listed in Table 2.

**Table 2:** Clinico-demographic details of patients with NSTI (*N* = 171)

Variable	Observation
Gender distribution [*n* (%)]	
Male	150 (87.7%)
Female	21 (12.3%)
Age distribution [*n* (%)]	
< 30 years	7 (4.1%)
31–50 years	46 (26.9%)
51–70 years	94 (55%)
> 70 years	24 (14%)
Distribution of comorbidities [*n* (%)]	
Diabetes mellitus	99 (57.9%)
Hypertension	76 (44.4%)
Ischemic heart disease	12 (7.01%)
Chronic liver disease	7 (4.09%)
Peripheral occlusive arterial disease	3 (1.75%)
Site of involvement [*n* (%)]	
Left foot	24 (14%)
Right foot	16 (9.4%)
Left leg	24 (14%)
Right leg	28 (16.4%)
Left lower limb	29 (17%)
Right lower limb	29 (17%)
Scrotum	16 (9.4%)
Right and left upper limb	5 (3%)
Presenting symptoms [*n* (%)]	
Pain	171 (100%)
Swelling	168 (98.2%)
Fever	90 (52.6%)
Signs at presentation [*n* (%)]	
Erythema/ discolouration	171 (100%)
Soft-tissue oedema	170 (99.4%)
Bullae	158 (92.4%)
Vital signs on examination [*n* (%)]	
Pulse rate (≥ 90 beats/min)	70 (40.9%)
Hypotension Systolic blood pressure (< 90 mmHg)	10 (5.8%)
Afebrile (< 38.0 °C)	157 (91.8%)
Tachypnoea (> 20/min)	68 (39.8%)

**Fig. 1:**
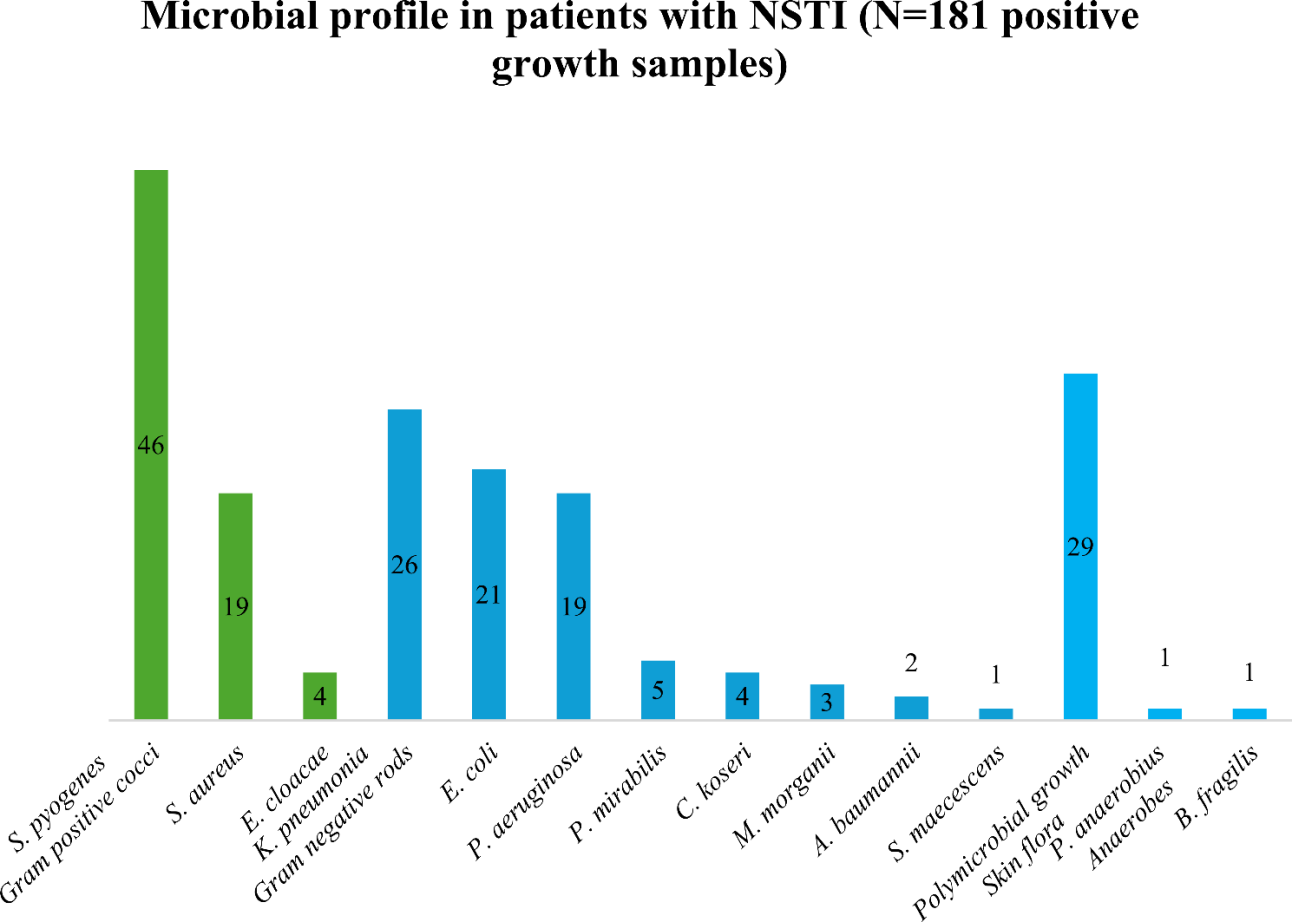
Microbial profile in patients with NSTI (*N* = 181 positive growth samples)

A total of 324 culture samples were collected during the study. The maximum number of times a culture sample collected from a patient was 4 times. 181 of the 324 samples sent indicated microbial growth. Significant growth (25.41%) was observed for *Streptococcus pyogenes*. One of our study’s patients showed anaerobic growth of *Bacteroides fragilis* and *Peptostreptococcus anaerobius*. Figure 1 depicts the microbiological profiles of the patients.

Of 181 positive wound culture growths, 141 showed antibiotic sensitivity. Ceftriaxone sensitivity was seen in 41/141 (29.1%) patients, whereas Amoxicillin-Clavulanic acid sensitivity was seen in 30/141 (21.3%) patients. Amikacin 13/141 (9.2%), Sulphamethoxole-Trimethoprim 14/141 (9.9%), and Ciprofloxacin 24/141 (17.02%) were among the other antibiotics tested. Teicoplanin was observed in five cases, and Piperacillin-Tazobactam and Clindamycin were observed in six cases. Meropenem (3/141), Gentamycin (2/141), Tinidazole, and Metronidazole sensitivity were observed in a single patient. Wound cultures that were negative for growth were mostly obtained on the second attempt, indicating the effectiveness of the empirical regime. However, a negative culture result alone would not be considered sufficient evidence to terminate ongoing antibiotic therapy. Overall clinical improvement and the residual debrided wound were always factored into decision-making and were considered better adjuncts in assessing the effectiveness of the healing process.

Seventy-two patients (42.1%) underwent radiography of the affected region at admission. Since CT scans delay primary surgical care, none of the patients underwent this procedure. Of the 72 patients who underwent X-ray imaging, 71 (98.6%) had gas shadows and soft tissue edema visible on the images. Procalcitonin levels were checked in 108 of the 171 patients with NSTI diagnoses at admission. Of these, 97/108 (89.8%) received a positive result (> 0.5ng), signifying sepsis and necessitating a blood culture for specific antibiotic treatment. 11/108 (10.2%) had a low procalcitonin level (< 0.5ng).

On admission, the LRINEC score was determined for each patient. A score ≥ 8 was obtained in 118/171 (69%) patients, suggesting a higher severity of the condition and a significant risk for NSTI. Seventeen (9.9%) patients had a score ≤ 5, while 36/171 (21.1%) had a score between 6 and 7 (Table 3).

**Table 3:** LRINEC scoring and severity among patients *n* = 171

Variable	Observation
LRINEC Score [*n* (%)]	
< 5	17 (9.9%)
6–7	36 (21.1.%)
≥ 8	118 (69%)

**Fig. 2:**
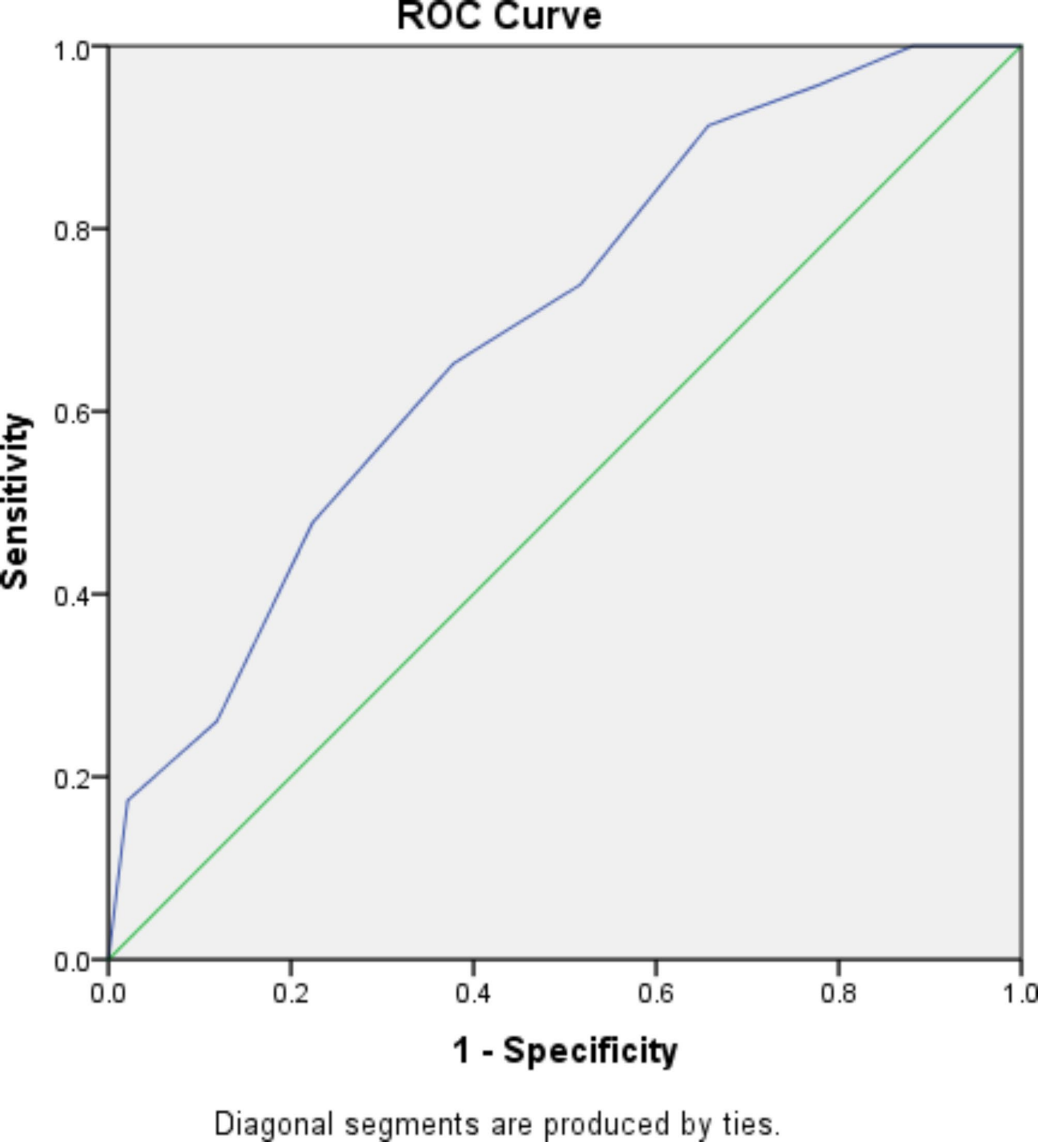
Receiver operating characteristic (ROC) curves for LRINEC for predicting NSTI

We constructed ROC curves to establish a suitable threshold for the diagnostic accuracy of the LRINEC score in the NSTI (Fig. 2). The Area Under the Curve was 0.694 (sensitivity: 95.7%; specificity: 77.6%). Thus, the area under the curve (AUC) was significantly greater than 0.5, which can be considered accurate for diagnosis.

The surgical details are presented in Table 4. Of the 171 patients included in our study, 169 underwent debridement at the time of admission. Ninety-six patients underwent definitive surgery involving reconstruction and amputation.

**Table 4:** Surgical description of the study population (*N* = 171)

Variable	Observation
Debridement carried out [*n*(%)]	
Debridement done	169 (98.9%)
Debridement not done	2 (1.2%)
Number of debridement [*n*(%)]	
No debridement (due to death)	2 (1.2%)
One debridement	87 (50.9%)
Two debridement	62 (36.3%)
Three debridement	18 (10.5%)
Four debridement	2 (1.2%)
Time taken for surgical debridement [*n*(%)]	
Median [IQR] time taken for initial debridement (in hours)	12 [6–48] hours
Less than 6 h	18 (10.5%)
6–12 h	59 (34.5%)
12–24 h	8 (4.7%)
More than 24 h	84 (49.1%)
Definitive surgery [*N* = 96] [n(%)]	
Skin graft	45 (26.3%)
Secondary suturing	13 (7.6%)
Ray amputation	13 (7.6%)
Above knee amputation	9 (5.3%)
Below knee amputation	16 (9.4%)
Duration of hospital stay [*n*(%)]	
Less than seven days	52 (30.4%)
7–15 days	55 (32.2%)
15–30 days	48 (28.1%)
More than 30 days	16 (9.4%)

Of the 171 patients diagnosed with NSTI, 143/171 (83.6%) had a complete recovery and were allowed to go home. 28/171 (16.4%) of the patients had succumbed to death.

The association between the LRINEC score and various parameters was assessed. The study found that the higher the LRINEC score, the statistical increase in mortality was noted on a chi-square test (*p* = 0.04). Hence, a cutoff of ≥ 6 had a high sensitivity of 95.7% in predicting mortality. Elevated procalcitonin levels also had a higher mortality (*p* = 0.031) in the chi-square test. However, no statistical association was noted between the number of days of hospitalization (*p* = 0.755) and the type of definitive surgery opted for (*p* = 0.14). With the objective of finding an association between mortality and surgical variables, 87.1% of the patients who underwent surgery recovered well, which was statistically significant (*p* = 0.008). Furthermore, 82.1% of them who had undergone surgery in less than 12 h recovered well; this association was statistically significant (*p* = 0.005).

**Fig. 3:**
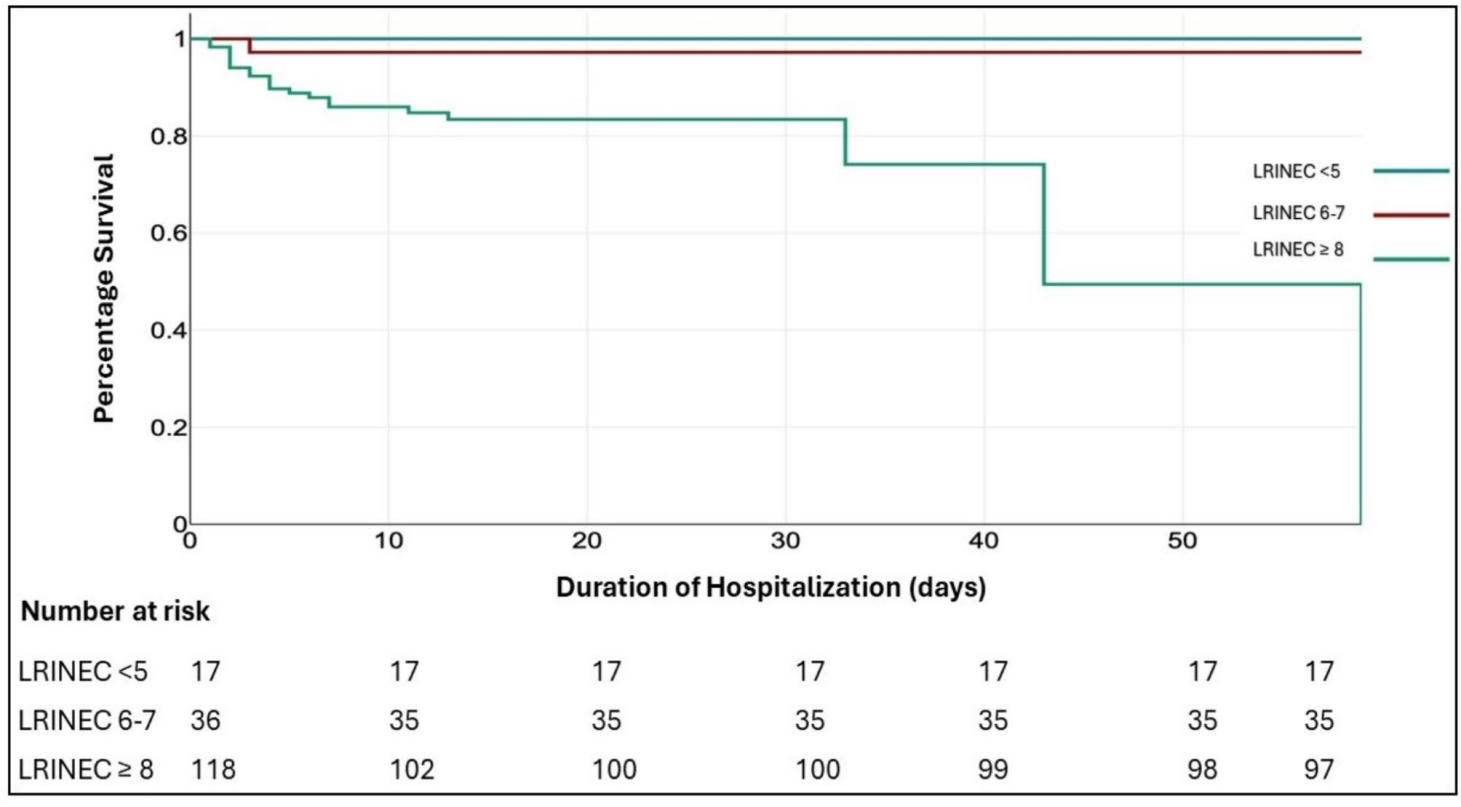
Kaplan- Meier survival curve according to the severity of LRINEC score. Group 1 -Low risk (< 5), Group 2 – Moderate risk (6–7), Group 3 – High risk (≥ 8)

Mortality in our patients was the maximum among the high-risk LRINEC score group (≥ 8) (17% [21/118]) Fig. 3.

A Kaplan-Meier survival curve was plotted to correlate the number of mortalities with the LRINEC score. From the graph, we can understand that the patients with a LRINEC score ≥ 8 had a higher hazard as compared to those with a lower LRINEC score. Also, mortality was higher in the initial 10 days of hospital admission.

## Discussion

NSTIs are rare but life-threatening diseases associated with high mortality and morbidity rates. The high morbidity and mortality rates associated with NSTIs underscore the need for early diagnosis and identification of potential risk factors for adverse outcomes. In the present study, we attempted to enumerate the clinical findings, demographics, and comorbidities of patients with NSTI.

The study involved 171 patients diagnosed with NSTI at our institution over 21 months. There were 150 (87.7%) male patients, indicating male preponderance.

Uncontrolled DM remains a predisposing factor in approximately 40–60% of cases in published data [13]. In the current study, approximately 57.1% of patients had poorly controlled diabetes. The most common comorbidity among patients with NF is diabetes mellitus, which is associated with longer hospital stays and higher death rates. Individuals with a history of diabetes mellitus showed a rapid increase in both mortality and NF severity. The hyperglycemic state may partially explain this result, which impairs immunity and promotes bacterial proliferation. The other prevalent comorbidity in this study was hypotension, which may result in reduced antimicrobial delivery, tissue oxygenation, and disruption of the microvascular supply [14].

The classic and frequent manifestations associated with NF usually include pain, tender local swelling, and fever. Of the 171 patients, all presented with pain, 168 (98.2%) with limb edema, and 90 (52.6%) with fever. Other clinical features included erythema in 171 patients, soft-tissue edema in 170 (99.4%), bullae in 158 (92.4%), hypotension in 101 (59.1%), and tachycardia in 161 (94.2%) patients. Goh et al. showed that 88% of the patients had edema, 79% had pain, and 77% had erythema when they first presented clinically. Due to the non-specific nature of these traits, over 75% of the patients had an initial misdiagnosis [6].

The clinical value of bacterial examination in the management of infections is substantial. Anaerobic and aerobic bacterial infections are typical sources of NSTI. The present study highlighted that the most common organisms isolated were *Streptococcus pyogenes* (25.4%), *Klebsiella pneumoniae* (14.3%), and *Escherichia Coli* (11.6%), which were the predominant gram-negative microorganisms. Wong et al. found that the most frequent cause (53.9%) was polymicrobial synergistic infection, with Enterobacteriaceae spp. and Streptococci spp. being the most frequently isolated microorganisms. Monomicrobial NSTI was mostly caused by Group A Streptococcus [15].

Unfortunately, the first stage of NSTI is frequently masked by non-specific manifestations, which prevents effective and timely specific therapy. Consequently, it is crucial to identify and diagnose patients as soon as possible and not depend solely on clinical symptoms. Seventy-two patients underwent X-ray imaging of the afflicted region; of these, 71 (98.6%) exhibited gas shadows and soft tissue edema, indicating a conclusive diagnosis of NSTI.

Of the 171 patients included in our study, 169 (98.9%) underwent surgical debridement. The patients underwent surgery after the initial evaluation and resuscitation. NSTI requires complex therapy consisting of early and repeated surgical debridement, broad-spectrum antimicrobial drugs, and intensive care treatment. 77 (45.5%) patients underwent surgical debridement within 12 h. 92 patients underwent debridement after 12 h, mostly due to logistical delays and the need for optimization of patients who were quite sick before surgery. These data suggest that early surgical intervention is crucial in reducing morbidity and mortality in patients with NSTIs. There still needs to be a clear definition of how early we should be. Kobayashi et al. reported significantly lower mortality in the early intervention group (within 12 h after diagnosis) [16]. Elliot and colleagues demonstrated in a group of 198 patients that survivors experienced a reduced duration between admission and first debridement (1·2 versus 3·1 day) [17]. Similarly, in a group of 89 patients, Wong et al. demonstrated that a delay of > 24 h prior to surgery was associated with a higher death rate (relative risk 9·4; *p* < 0·05) [15].

NSTIs are rare, but rapidly progressive and potentially lethal bacterial diseases. In our study, 143 (83.6%) patients recovered from the debilitating disease and were discharged home, and 28 (16.4%) patients died. Mortality was significantly lower in patients who underwent early surgical debridement than in those with a delay in surgical treatment (41% vs. 58%, *p* ≤ 0.005). Elliot et al. reported a mortality rate of 25.3% in 198 patients [17]. A retrospective study conducted in Thailand by Khamnaun et al. included 1,504 patients with a 19.3% death rate [18].

A study by Kurian et al. demonstrated several key similarities and differences. Our study revealed a higher prevalence of NF in male patients, with 87.7% compared to 65% in their study. Type 2 Diabetes mellitus emerged as the predominant comorbidity in both investigations, affecting 57.1% of patients in our study and 66% in theirs. Microbial profiles showed similarities; in our study, 25.41% of infections were attributed to Streptococcal organisms, compared to 19% in their study. Moreover, 10.49% of our cases involved Staphylococcal organisms, whereas they reported 16%. Both studies emphasized the critical role of early surgical intervention for improved prognosis, with Kurian et al. reporting a mortality rate of 34% attributed to delayed presentation and intervention [19].

Similarly, Prabhu et al., in a study conducted at a rural tertiary center, observed a predictive association of NF in patients aged over 40 years, consistent with our findings. Both studies noted a predominance of male patients, with Prabhu et al., reporting 76% compared to our 87.7%. Common symptoms across both studies included pain, swelling, and fever. Type 2 Diabetes mellitus was identified as the primary comorbid factor, affecting 52% of patients in Prabhu et al.’s study and 57.9% in ours. Regarding microbiological profiles, both studies identified Streptococcal species (70%) as the most prevalent gram-positive causative organism. However, Prabhu et al. reported E.coli (74%) as the most common gram-negative species, whereas our study identified Klebsiella pneumoniae (14.36%) more frequently. Extremities were identified as the most affected site in both studies [20].

In a study by Tarrino et al., which evaluated the utility of the LRINEC score in a tertiary hospital similar to ours, the median LRINEC score among 45 cases was 7. Their study found that 60% (27 patients) had an LRINEC score ≥ 6, whereas our study indicated that 90% (154 patients) had an LRINEC score ≥ 6. Both studies highlighted high mortality rates, prolonged hospital stays, and increased amputation rates among the high-risk group (LRINEC score ≥ 6) [21].

The LRINEC score was calculated for all the patients at the time of admission. Among the 171 patients, 154 (90%) had an LRINEC score of ≥ 6. At an LRINEC cutoff score of 6, the model had a sensitivity of 95.7%. In a study conducted by Wong et al. at an LRINEC score cutoff of 6, the model had a positive predictive value of 92.0% [15]. The AUC in the ROC for our model was 0.694 (95% confidence interval 0.584–0.804) in this study. The curve represents the relationship between the corresponding values of sensitivity and specificity with all possible values of probabilities as a cutoff point to predict the presence of an NSTI.

The limitations of this study include its single-center design, limited sample size, and lack of sample size estimation. To obtain more precise statistics, multicenter research and an increase in sample size should be conducted. The heterogeneity of NF and healthcare facilities will prevent drawing useful inferences if a multicenter study includes non-homogeneous patient groups. Additionally, the design and funding of large multicenter prospective studies are challenging but can offer better insights into improving NF patient outcomes. There was no comparison of the therapeutic effects in this study, nor was there a control group. Establishing a control group in an observational study like ours is challenging. Performing a randomized controlled trial may be questioned on ethical grounds, as neither the extent of surgical procedures nor the specific antibiotics can be predetermined in a polymicrobial, rapidly progressive, life-threatening infection like NF. We were unable to compare the therapeutic results with alternative treatment modalities; instead, we could only objectively summarize the early characteristics and causal aspects of NSTI and disease therapy.

## Conclusion

Necrotizing soft tissue infections (NSTIs) are rare but rapidly progressing and life-threatening conditions with high morbidity and mortality rates. Our study emphasizes the critical importance of early recognition, accurate diagnosis, and prompt surgical intervention. In our study, the LRINEC score with a cutoff of 6.0 or more and increased procalcitonin level were predictors of death in patients with NSTI. Early and thorough surgical debridement is the single most crucial intervention of choice in patients with NF, regardless of disease severity and the LRINEC score. Patients who underwent surgical debridement within 12 h of diagnosis had significantly better survival rates, underscoring the importance of urgent surgical management in patients with NF.

## Data Availability

The data supporting this study’s findings are available upon request from the corresponding author (AR). The data are not publicly available because they contain information that could compromise the privacy of research participants.
